# The comparative anti-cancer effects of krill oil and oxaliplatin in an orthotopic mouse model of colorectal cancer

**DOI:** 10.1186/s12986-022-00646-8

**Published:** 2022-03-02

**Authors:** Abilasha Gayani Jayathilake, Majid Hassanzadeganroudsari, Valentina Jovanovska, Rodney Brain Luwor, Kulmira Nurgali, Xiao Qun Su

**Affiliations:** 1 Institute for Health and Sport, Victoria University, P.O. Box 14428, Melbourne, 8001 Australia; 2 Department of Surgery, The Royal Melbourne Hospital, The University of Melbourne, Parkville, Australia; 3 Department of Medicine-Western Health, The University of Melbourne, Melbourne, Australia; 4 Regenerative Medicine and Stem Cells Program, Australian Institute for Musculoskeletal Science (AIMSS), Melbourne, Australia

**Keywords:** Krill oil, Oxaliplatin, Orthotopic model of colorectal cancer, Caspase 3/9, PD-L1/PD-L2, HSP-70

## Abstract

**Background:**

Our in vitro studies demonstrated that krill oil (KO) has anti-cancer potential. This study aimed to compare the anti-cancer effects of KO with a commonly used chemotherapeutic drug, oxaliplatin and to identify the molecular mechanisms associated with KO supplementation in a mouse model of colorectal cancer (CRC).

**Methods:**

Thirty-six male Balb/c mice were randomly divided into six groups. Five groups received standard chow diet supplemented with KO (150 g/kg)), corn oil (150 g/kg), KO combined with ½ dose of oxaliplatin (1.5 mg/kg body weight/3 times per week), corn oil combined with ½ dose of oxaliplatin (1.5 mg/kg body weight/3 times per week), or a full dose of oxaliplatin (3 mg/kg body weight/3 times per week). The control (sham) group received a standard chow diet. Treatments started three weeks before and continued for three weeks after orthotopic CRC induction. The number of metastases, tumour weight and volume were quantified *ex-vivo*. The expression of cytochrome *c*, cleaved caspase-9 and -3, DNA damage, PD-L1, PD-L2 and HSP-70 were determined.

**Results:**

A significant reductions in the weight and volume of tumours were observed in mice treated with KO and KO plus a ½ dose of oxaliplatin compared to the sham group, similar to oxaliplatin-treated mice. KO, and KO plus ½ dose of oxaliplatin significantly increased the expression of cytochrome *c,* cleaved caspase-9 and -3, and DNA damage and decreased expression of PD-L1, PD-L2 and HSP-70 in tumour tissues compared to the sham group.

**Conclusions:**

The in vivo anti-cancer effects of KO are comparable with oxaliplatin. Thus, dietary KO supplementation has a great potential as a therapeutic/adjunctive agent for CRC treatment.

## Background

Colorectal cancer (CRC) is the third most common cancer and the fourth leading cause of cancer-related mortality worldwide, with almost 1.8 million new cases and 881,000 deaths reported in 2018 [1]. A complete resection of the primary tumor would be effective to cure CRC diagnosed at an earlier stage. However, CRC is often asymptomatic at an early stage, and most cases are identified at a late stage. Approximately 56% of patients die from CRC due to distant metastases present in approximately 25% of patients at the initial diagnosis [2–4]. Currently, the advanced stage of CRC is treated with chemotherapy, radiotherapy, their combination, or adjuvant pre- or post-surgical chemotherapy. Commonly used chemotherapeutic agents are 5-fluorouracil (5-FU), oxaliplatin, irinotecan, or their combinations. Unfortunately, these treatments associate with numerous acute and long-term side effects. Nearly 85% of patients reduce the dose or discontinue the treatment due to these side effects [5].

Oxaliplatin is a third-generation platinum-based anti-cancer chemotherapeutic drug primarily used as a first-line treatment for metastatic colon or rectum cancer. The side effects associated with oxaliplatin treatment include peripheral neurotoxicity, nephrotoxicity, cardiotoxicity, and several gastrointestinal (GI) complications such as diarrhoea, nausea, abdominal pain, vomiting, and anorexia [6]. The symptoms can continue for up to 10 years after treatment has stopped [7, 8].

Epidemiological and experimental findings have shown that natural products containing a high level of long-chain n-3 polyunsaturated fatty acids (LC n-3 PUFA), mainly eicosapentaenoic acid (EPA) and docosahexaenoic acid (DHA), such as fish oil, are potentially effective adjuncts for cancer treatment with minimal or no side effects [9]. Krill oil (KO), extracted from Antarctic krill (*Euphasia superba*), is also a rich source of LC n-3 PUFA. It is different from fish oil since EPA and DHA in KO are mainly bound to phospholipids instead of triglycerides as in fish oil [10, 11]. This suggests the potentially higher bioavailability of KO-derived LC n-3 PUFA compared to fish oil. In addition, KO contains the antioxidant astaxanthin, vitamins, and minerals [11]. Several animal studies and human trials have reported various health benefits of KO, including its anti-inflammatory properties [12] and its therapeutic effects on glucose metabolism [13], brain disorders [14], cardiovascular diseases [15], as well as CRC [16].

Zhu et al. [17] and Su et al. [18] reported the anti-proliferative properties of KO on CRC and osteosarcoma cells. Our previous in vitro studies demonstrated that free fatty acid extract (FFAE) of KO inhibits the proliferation and induces the death of CRC cells through alteration of mitochondrial membrane potential, leading to DNA damage and apoptosis [19, 20]. Furthermore, Zhen et al. [21] found that EPA and DHA of KO possess a special E-configuration associated with a high anti-proliferative activity observed in several types of cancer cells.

Programmed death-ligands (PD-Ls), PD-L1 and PD-L2, expressed on the surface of tumour cells play a major role in suppressing the immune response and, therefore, providing an immune escape mechanism for cancer progression. The binding of PD-Ls to a programmed cell death protein 1 (PD-1), an immune checkpoint protein expressed on activated T cells, B cells, and myeloid-derived dendritic cells, initiates immunosuppressive signalling pathways [22, 23]. The interaction between the PD-1 with PD-Ls inhibits T cell function, and therefore, provides a favourable environment for the tumour cells to evade immune response [22, 23]. Our previous study found that the FFAE of KO suppresses PD-L1 expression in CRC cells [24]. Heat shock proteins (HSPs) are highly preserved molecular chaperones found in all living organisms. They play different roles in the various cellular processes [25]. HSP-70 is a powerful anti-apoptotic protein that suppresses mitochondrial damage and DNA fragmentation by reducing caspase activation [26, 27]. Overexpression of HSP-70 increases tumorigenicity and promotes cancer cell growth [28, 29]. Upregulation of HSP-70 correlates with the advanced clinical stages, metastatic spread, and poor survival of CRC patients [30], indicating that HSP-70 may be a potential therapeutic target for CRC treatment. Cai et al. [31] have demonstrated that treatment with DHA in combination with celecoxib inhibits prostate cancer cell growth by mediating protein–protein interactions between HSP70 and p53. However, Chung et al. [32] showed no change in HSP-70 level in prostate cancer cells following n-3 PUFA treatment.

There is little information available on the anti-cancer properties of KO in vivo and the associated molecular mechanisms. A previous study showed that KO treatment inhibits tumour growth in mice bearing human colon cancer xenografts, but the mechanisms of its anti-cancer effects have not been determined [16]. The aims of this study are to (i) investigate the effect of KO supplementation on CRC tumour growth and metastasis in comparison with oxaliplatin treatment; (ii) determine whether the in vivo anti-cancer effect of KO is associated with the mitochondrial death pathway, and (iii) investigate the in vivo effect of KO on the expression of PD-L1, PD-L2, PD-1, and HSP-70. To the best of our knowledge, this is the first animal study comparing the efficacy of KO supplementation with oxaliplatin on CRC growth and metastasis, and investigating the potential molecular targets of KO.

## Material and methods

### Cell culture

The CT-26 mouse colon adenocarcinoma cell line was obtained from the American Type Culture Collection (ATCC), Manassas, VA, USA (Catalogue No. CRL-2638). Cells were cultured in RPMI1640 medium (Sigma Aldrich, Castle Hill, NSW, Australia) supplemented with fetal calf serum (FCS, 10%) (Hyclone Quantum Scientific, Clayton South, VIC, Australia) and 4-2-hydroxyethyl-1-piperazineethanesulfonic acid, glutamine (10 mM), penicillin (100 U/mL)/streptomycin (100/mL) and sodium pyruvate (10 mM) (Sigma Aldrich, Castle Hill, NSW, Australia). Cells were maintained in a humidified atmosphere in 5% CO_2_, 95% air at 37 °C. More than 90% of viable cells obtained after trypsinization of the monolayer culture were used to make the injection. The cell viability was ensured by the trypan blue exclusion assay.

### Experimental model

The animal study was approved by the Victoria University Animal Ethics Committee (AEC No.17/008). The mice were maintained in accordance with the guidelines of the Australian National Health and Medical Research Council Code of Conduct on the care and use of laboratory animals for scientific purposes. Balb/c mice (n = 36, 6–7 weeks old, weighted 18–25 g) were obtained from the Animal Resource Center (Perth, Australia). Upon arrival, animals were randomized into six groups of six mice in each [sham (control) group and five treatment groups] and housed in OptiMouse cages under pathogen-free conditions with a 12 h light/dark cycle in a well-ventilated room at 22 °C with free access to food and water.

### Treatments

**Fig. 1:**
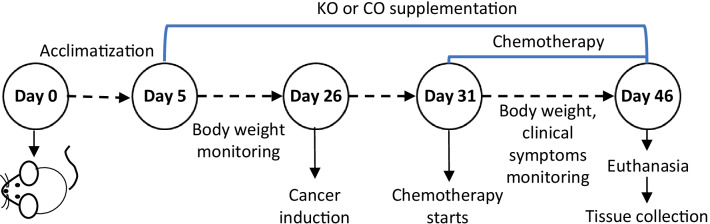
The schematic overview of study design

At the end of the 5-day acclimatization period, treatments were introduced 3 weeks before CRC induction and continued for 3 weeks post-surgery (Fig. 1). Group 1 (sham) was fed with a standard rodent diet (manufactured by Specialty Feeds, Western Australia) prior to and after cancer induction. Group 2 (positive control) received a rodent diet supplemented with 150 g/kg of corn oil (Gold Fish Brand, Australia) prior to and after cancer induction. Group 3 (positive control) was given a rodent diet supplemented with 150 g/kg of corn oil prior to cancer induction and the same concentration of corn oil combined with intraperitoneal injections of ½ dose of oxaliplatin (1.5 mg/kg, Tocris Bioscience, UK) 3 times/week starting at day 5 post-surgery for 2 weeks. Group 4 received a rodent diet supplemented with 150 g/kg of KO (Swisse Wellness Pty Ltd., Victoria, Australia) prior to and after cancer induction. Group 5 was supplemented with 150 g/kg of KO prior to cancer induction and the same concentration of KO combined with intraperitoneal injections of ½ dose of oxaliplatin 3 times/week starting at day 5 post-surgery for 2 weeks. Group 6 received a standard rodent diet and intraperitoneal injections of a full dose of oxaliplatin (3 mg/kg) 3 times/week starting at day 5 post-surgery for 2 weeks. Groups 1, 2 and 4 received intraperitoneal injections of 200 µL of sterile saline 3 times/week starting at day 5 post-surgery for 2 weeks. KO contains 13% EPA, 7.5% DHA and 0.03% Astaxanthin. The percentage of KO supplemented in the feed (150 g/kg) was determined as the most effective dose based on the pilot study results (unpublished data). The amount of oil provided to animals was calculated based on their feed consumption rate (g/day) and adjusted regularly. Oil supplement was mixed with the standard rodent diet by loading into a pre-calibrated hole in the pellet. KO and corn oil containing diets were prepared fresh and replaced daily to minimize the oxidation of polyunsaturated fatty acids. The consumption of feed was measured daily, and body weight was recorded twice a week.

### Orthotopic CRC induction

At the end of 3-week treatment, CT-26 cells (1 × 10^6^ cells in 25 µL Matrigel, Sigma, Australia) were injected into the caecum of mice to induce cancer as described before [33]. After 21 days of tumour inoculation, or when the tumour size was over 1 cm^3^, the animals were culled by overdosing with pentobarbitone (100 mg/kg). Kaplan Meier survival analysis was used to assess the survival rate of animals in each group. Immediately after mice were sacrificed, their body was dissected and metastases were identified via visual observation. The number of tumours were counted and recorded in all organs including skin and muscles. The tumour samples were immediately removed. Approximately 1/3 of tumour tissues were snap-frozen in liquid nitrogen and stored at − 80 °C until further analysis by Western blotting, remaining 2/3 of tumour tissues were incubated overnight in Zamboni’s fixative [2% formaldehyde, 0.2% picric acid, and 0.1 M sodium phosphate buffer (pH 7)] at 4 °C for immunohistochemical or histological analysis.

### Histology

Zamboni’s fixed tissues were washed 3 × 10 min with dimethyl sulfoxide (DMSO) (Sigma, Australia) and 3 × 10 min with phosphate-buffered saline (PBS). Tissues were embedded in paraffin, sectioned at 5 μM and used for a standard Haematoxylin and Eosin (H&E) staining. Initially, sections were immersed in xylene (3 × 4 min), followed by 3 min in 100% ethanol, 2 min in 90% ethanol, 2 min in 70% ethanol, and washed with tap water. Then they were immersed for 4 min in Harris Haematoxyline (Richard Allan Scientific, USA), rinsed with tap water, followed by 1 min in Scott’s tap water, immersed in 1% Eosin (Amber Scientific, Australia) for 3 min, rinsed in tap water, 100% ethanol (2 × 1 min), xylene (2 × 3 min), and mounted on the distyrene plasticizer xylene (DPX) with a coverslip. The histological slides were observed under a fluorescent microscope. Each slide was analysed by randomly capturing 10 sections per preparation at 20 × magnification using a DP72 camera and processed using cellSens standard 1.4.1 software (Olympus, Australia). The tumour cells counterstained with Haematoxylin stain were quantified within an area of 2 mm^2^ using the Image J software (National Institute of Health, USA). Five different histological samples from each mouse were examined and the number of tumour cells was averaged to obtain the final results. All images were analysed blindly.

### Immunohistochemistry

After 24 h of fixation in Zamboni’s solution, tumour tissues were washed with 100% DMSO (Sigma-Aldrich, Australia) 3 × 10 min to remove the fixative, and this was followed by 3 × 10 min washing with PBS. Then samples were embedded in an Optimum Cutting Temperature compound (OCT Sakura, Tissue Tek, USA) and sectioned at a thickness of 10 µM using a cryostat (Leica CM 1950, Biosystems, Germany). The tissues were mounted onto microscopic slides (Superfrost^R^ Plus, Thermo Fisher Scientific, Australia) and incubated with 10% donkey serum for 1 h at room temperature to block the endogenous peroxidase activities. Following the washing with PBS, tumour sections were incubated overnight at room temperature in a humidified atmosphere with primary antibodies for cytochrome *c* (1:1000, rabbit mAb, Abcam 133,504 [EPR 1327], USA), cleaved caspase-9 (1:500, rabbit mAb, Asp 330 [E5Z7N], Cell Signaling Technologies, USA), cleaved caspase-3 (1:500, rabbit mAb, Asp175 [5A1E], Cell Signaling Technologies, USA), DNA/RNA damage (1:500, anti-8-OHdG mouse mAb [15A3], Abcam, USA), PD-L1 (1:500, rabbit mAb, [ab213480], Abcam, USA), PD-L2 (1:500, rabbit, ab187662, Abcam, USA), PD-1 (1:500, rat [clone RPM1-14], Bio-Cell, USA), HSP-70 (1:500, mouse mAb, ab2787, Abcam, USA). On the following day, the slides were washed 4 × 10 min with PBS-T (PBS + 0.1% Tween-20) and incubated with secondary antibodies (diluted to 1:250) labeled with different fluorophores: Alexa Fluor 594 and 488 conjugated donkey anti-rabbit (Jackson Immuno Research Laboratories, USA), Alexa Fluor 488-conjugated donkey anti-mouse (Jackson Immuno Research Laboratories, USA) and Alexa Fluor 594-conjugated donkey anti-rat (Jackson Immuno Research Laboratories, USA) at room temperature for 2 h. This was followed by 4 × 10 min washes with PBS-T. Then the slides were incubated with a fluorescent nucleic acid stain, 4′,6′-diamidino-2-phenylindole dihydrochloride (DAPI, 14 nM, Life Technologies, Australia) for 2 min. Finally, all slides were washed with PBS for 10 min and cover-slipped using a fluorescent mounting medium (DAKO, Australia). Images were taken with the Eclipse Ti confocal laser scanning system (Nikon, Tokyo, Japan). The excitation wavelengths for FITC and Alexa Fluor 594 were adjusted to 488 nm and 559 nm, respectively. Each fluorophore was measured using 8 images taken at 40 × magnification with a total area of 1 mm^2^. All images were then calibrated to standardize for a minimum basal fluorescence and converted to binary. Fluorescence intensity was measured using Image J software (National Institute of Health, USA). The results were verified through at least three individual repeated experiments. All slides were coded, and analysis was performed blindly.

### Western blot

Frozen pooled tumour samples (n = 6 mice/group) were homogenized using a Polytron homogenizer (Kinematica AG, Switzerland) for 15 s in ice-cold radioimmunoprecipitation assay (RIPA) buffer (pH 7.4, 150 mM NaCl, 0.1% SDS, 0.5% sodium deoxycholate, 1% NP-40 in PBS, Sigma, Australia) containing a protease and phosphatase inhibitors cocktail (Roche Applied Science, USA). The lysis was centrifuged at 12,000 rpm for 20 min at 4 °C and supernatants were used for western blot analysis. Protein concentration was determined by the Pierce bicinchoninic acid (BCA) assay (Thermo Fisher Scientific, Australia). Protein samples were loaded as 12 µg/lane onto Mini-PROTEAN® TGX™ (4–20%) stain-free precast gel (Bio-Rad, USA) and separated by 10% sodium dodecyl sulfate polyacrylamide gel electrophoresis (SDS-PAGE). The separated fragments were transferred to 0.22 µm polyvinylidene fluoride membranes, which were blocked with 5% skim milk in PBST (0.1% Tween-20) by incubating at room temperature at 40 rpm speed shaker for 90 min. Then the membrane was incubated with primary antibodies against cytochrome *c* (1:1000, rabbit, Abcam 133,504 (EPR 1327), USA), cleaved caspase-9 (1:1000, rabbit mAb, Asp 330 (E5Z7N), Cell Signaling Technologies, USA), cleaved caspase-3 (1:1000, rabbit mAb, Asp175 (5A1E), Cell Signaling Technologies, USA), DNA/RNA damage (1:500, mouse anti-8-OHdG mAb, (15A3), Abcam, USA), PD-L1 (1:1000, rabbit mAb, Cell Signaling Technologies, USA), PD-L2 (1:1000, rabbit, Cell Signaling Technologies, USA), and GAPDH as a control (1:2000 dilution, rabbit, Santa Cruz Biotechnology, USA) overnight at 4 °C. The membrane was washed four times in PBS-T (0.1% Tween-20) and incubated with horseradish peroxidase (HRP)-conjugated secondary antibodies, donkey anti-rabbit IgG (1:10,000, 31,458, Thermo Fisher Scientific, Australia), and horse anti-mouse IgG (1:10,000, Cell Signaling Technology, USA) for 2 h at room temperature. Again, the membrane was washed three times in PBS-T. To detect the protein levels, the membrane was incubated with clarity enhanced chemiluminescence reagents (Clarity™ Western ECL Substrate, Bio-Rad, USA) for 5 min. Chemiluminescence signals were captured using the FUSION FX System (USA). The expression level of each protein was quantified using Fusion Capt advanced FX7 software.

### Statistical analysis

All data were analysed using SPSS 22 software (IBM, USA). Mixed model ANOVA was used to determine the significance between treatments. The significance of repeated measures at different time points was analysed using one-way ANOVA. Post-hoc analysis was conducted using the Tukey HSD test for multiple comparisons. *P* < 0.05 was considered significant. The Kaplan Meier survival analysis was performed to assess the survival rate of animals in each group using the formula: Survival Rate = (number of subjects living at the start – number of subjects died)/ number of subjects living at the start. One-way ANOVA was performed to compare the survival rate of animals in different experimental groups. The results were expressed as mean ± SD in tables and mean ± SEM in figures.

## Results

### The effects of KO supplementation on mouse survival rate and tumour growth

**Fig. 2:**
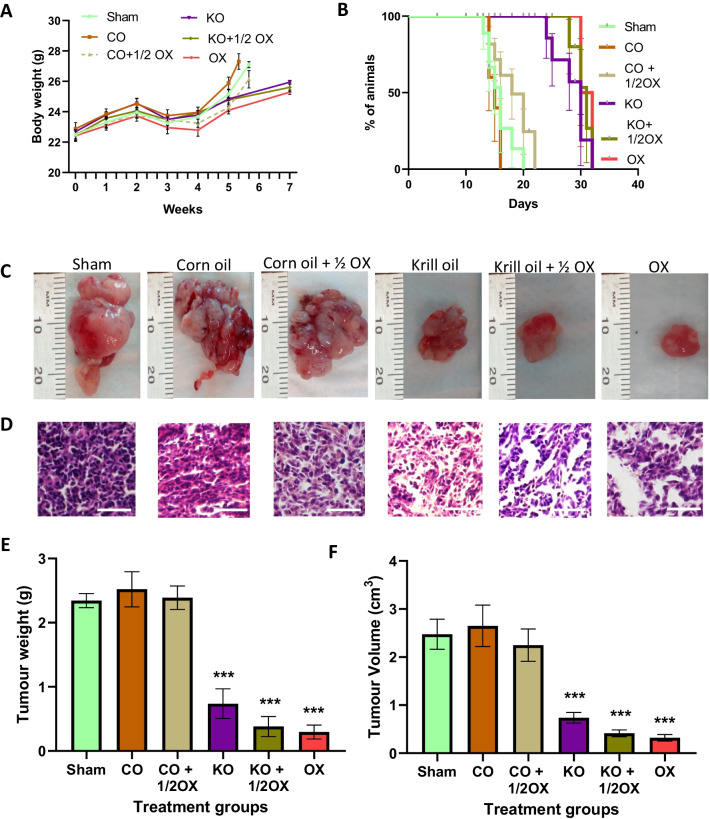
Effects of treatments on the body weight, survival rate, and tumour growth in Balb/c mice with orthotopic CRC. **A** The effects of treatments on the animal body weight. **B** Kaplan–Meier survival plot representing the percentage of survival of mice after treatment. **C** Representative images of orthotopic tumours excised from different treatment groups. **D** Representative histology images of tumour tissues (H&E stained, Magnification = 20X). **E** The mean tumour weight and **F** volume following various treatments. N = 6 mice per group. The results are expressed as mean ± SEM; ***P < 0.001 compared to the sham group

The effects of KO supplementation, alone or combined with a ½ dose of oxaliplatin, in Balb/c mice were compared to corn oil treatment groups (corn oil alone or combined with a ½ dose of oxaliplatin), sham (control) group, and a full dose of the oxaliplatin treatment group. No significant difference in the body weight was observed between any groups, although the oxaliplatin-treated group had the lowest body weight gain (Fig. 2A). There were no significant difference in food intake among treatment groups before or after CRC induction. The average food intake before CRC induction ranged between 2.4 and 2.5 g/day among the treatment groups, while after CRC induction it was 2.4 g/day for sham group, 2.2 g/day for both corn oil and corn oil + 1/2 oxaliplatin groups, 2.5 g/day for krill oil group, 2.6 g/day for krill oil + ½ oxaliplatin group, and 2.2 g/day for oxaliplatin group. The survival rate of animals supplemented with KO alone, KO combined with a ½ dose of oxaliplatin, and a full dose of oxaliplatin was significantly higher compared to the sham group (Fig. 2B). It was also noticed that the survival rate of animals from the two KO treatment groups (KO alone or in combination with ½ dose of oxaliplatin) was similar to the survival rate of animals treated with the full dose of oxaliplatin. Moreover, animals from KO supplemented groups did not show any noticeable side effects compared to the animals received the full dose of oxaliplatin which had a poor appetite, were lethargic, and less active.

The results of the histological examination of tumours collected from all groups (Fig. 2C) are presented in Fig. 2D. The number of tumour cells was significantly reduced following treatments with KO alone, KO combined with a ½ dose of oxaliplatin, as well as oxaliplatin alone, with a 53.5%, 58.8% and 53.9% decrease being observed respectively compared to the sham group. In contrast, no change in the number of tumour cells was observed in animals treated with corn oil, either alone or in combination with ½ dose of oxaliplatin compared to the sham group (Fig. 2D).

There were no significant changes in the total mean weight and volume of tumour between the two corn oil groups (corn oil alone and corn oil combined with a ½ dose of oxaliplatin) and the sham group at the end of the treatment period (*P* > 0.05) (Fig. 2E, F). In contrast, animals receiving KO treatment showed significantly reduced tumour weight (0.83 ± 0.2 g vs. 2.63 ± 0.2 g in the sham group and 2.73 ± 0.3 g in corn oil group) and volume (0.74 ± 0.1 cm^3^ vs. 2.5 ± 3.1 cm^3^ in the sham group and 2.7 ± 0.4 cm^3^ in the corn oil group) (*P* < 0.001 for both). These represent a 69.3% reduction in tumour weight and a 70.1% reduction in tumour volume compared to the sham group. Similarly, compared with the corn oil group, the tumour weight was reduced by 69.6%, and tumour volume by 72.1%. Further reduction was observed in animals received KO and KO combined with a ½ dose of oxaliplatin, with 0.42 ± 0.1 g being recorded for tumour weight and 0.4 ± 0.1 cm^3^ for tumour volume, indicating a reduction of 84.2% and 83.3%, respectively, compared to the sham group (*P* < 0.001 for both). For animals treated with a full dose of oxaliplatin, the average tumour weight and volume were 0.37 ± 0.1 g and 0.3 ± 0.1 cm^3^ respectively (*P* < 0.001 for both), indicating a reduction of 85.9% in tumour weight and 87.1% in tumour volume compared to the sham group (Fig. 2E, F). The differences between two KO -treated groups and a full dose of the oxaliplatin-treated group were not statistically different (*P* > 0.05), indicating that treatment with KO alone or KO combined with a ½ dose of oxaliplatin achieved similar outcomes as a full dose of oxaliplatin treatment.

### The effect of KO supplementation on tumour metastasis

Mice in the sham group showed an aggressive tumour growth and metastasis in the abdomen and various organs, including the small intestine, colon, liver, spleen, kidneys, and diaphragm. Mice from the groups received corn oil and corn oil with ½ dose of oxaliplatin also exhibited extensive tumour growth and metastasis, similar to the sham group. In contrast, two out of six mice treated with KO showed reduced metastasis compared to the sham animals. The most noticeable effects on metastasis were observed in animals treated with KO combined with a ½ dose of oxaliplatin, with only one out of six animals showing tumour metastasis and the remaining five animals having no detectable metastasis. Furthermore, the number of metastasized locations and the size of tumours were reduced significantly, similar to the oxaliplatin-treated group compared to the sham group.

### Expression of cytochrome *c*, caspase-9, caspase-3, and DNA damage in tumours following KO treatment

**Fig. 3:**
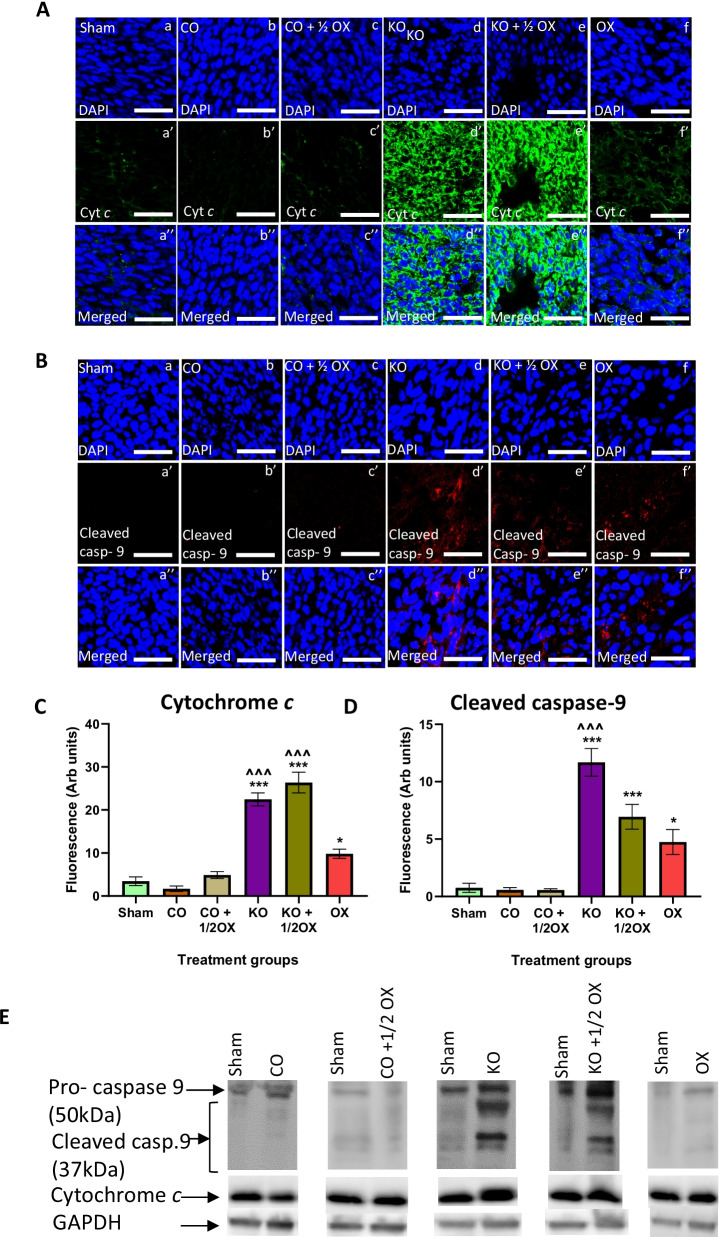
Expression of cytochrome *c* and cleaved caspase-9 following treatments. The expression of cytochrome *c* (**A**) and cleaved caspase-9 (**B**) in tumours following different treatments determined immunohistochemically using monoclonal antibodies. Quantitative analysis of the fluorescence intensity of cytochrome *c* (**C**) and cleaved caspase-9 (**D**) expression in tumour tissues following various treatments determined by immunohistochemistry. Scale bar = 50 µM. Magnification = 40X. N = 6 mice per group. The level of fluorescence was measured using 8 randomly taken images that provide a total area of 1 mm^2^. The results are expressed as mean ± SEM; *P < 0.05, ***P < 0.001 compared to the sham group; ^^^P  < 0.001 compared to the oxaliplatin-treated group. **E** The expression of cytochrome *c* and cleaved caspase-9 measured by western blotting in tumours from different treatment groups

Figure 3 shows the effects of treatments on the expression of cytochrome *c* and cleaved caspase-9 in tumour tissues. Animals treated with KO alone, KO combined with a ½ dose of oxaliplatin, and a full dose of oxaliplatin showed a significantly higher cytochrome *c* expression [61.3% (*P* < 0.001), 78.15% (*P* < 0.001), and 19.25% (*P* < 0.05), respectively] compared to the sham group (Fig. 3A, C). These three treatments also resulted in a significantly higher expression of cleaved caspase-9 in tumours with an increase of 85.2% (*P* < 0.001), 68.4% (*P* < 0.001), and 21.3% (*P* < 0.05), respectively (Fig. 3B, D). The results of the expression of cytochrome *c* and cleaved caspase-9 observed through western blotting (Fig. 3E) were consistent with the findings of the immunohistochemistry assay.

**Fig. 4:**
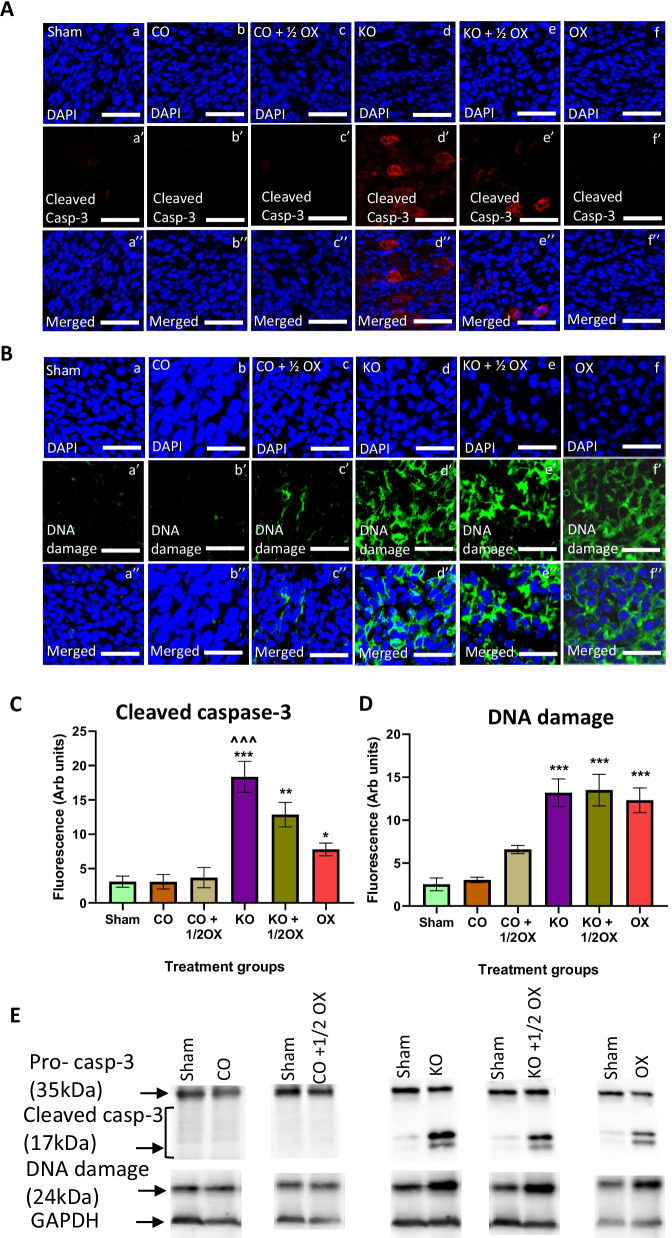
Expression of cleaved caspase-3 and DNA damage following treatments. The expression of cleaved caspase-3 (**A**) and DNA damage (**B**) in tumours following different treatments determined immunohistochemically using monoclonal antibodies. Quantitative analysis of the fluorescence intensity of cleaved caspase-3 (**C**) and DNA damage (**D**) expression in tumour tissues following various treatments determined by immunohistochemistry. Scale bar = 50 µM. Magnification = 40X. N = 6 mice per group. The level of fluorescence was measured using 8 randomly taken images that provide a total area of 1 mm^2^. The results are expressed as mean ± SEM; *P < 0.05, **P < 0.01, ***P < 0.001 compared to the sham group; ^^^P < 0.001 compared to the oxaliplatin-treated group. **E** The expression of cleaved caspase-3 and DNA damage measured by western blotting in tumours following various treatments

Furthermore, KO treatment significantly increased the expression of cleaved caspase-3 by 92.93% compared to the sham group (*P* < 0.001) (Fig. 4A, C). Treatment with KO combined with a ½ dose of oxaliplatin increased the expression of cleaved caspase-3 by 77.95% (*P* < 0.01), and the full dose of oxaliplatin by 41.73% (*P* < 0.05). DNA damage was also significant compared to the sham group, with an increase of the DNA damage resulting from the treatment with KO alone, KO combined with a ½ dose of oxaliplatin and a full dose of oxaliplatin by 48.3%, 62.4%, and 31.6%, respectively (*P* < 0.001 for all) (Fig. 4B, D). Results of immunohistochemistry assay for the expression of cleaved caspase-3 and DNA damage were further verified by western blotting (Fig. 4E), and consistent results were observed.

### Expression of PD-L1, PD-L2, PD-1 and HSP-70 in tumours following the treatment with KO

**Fig. 5:**
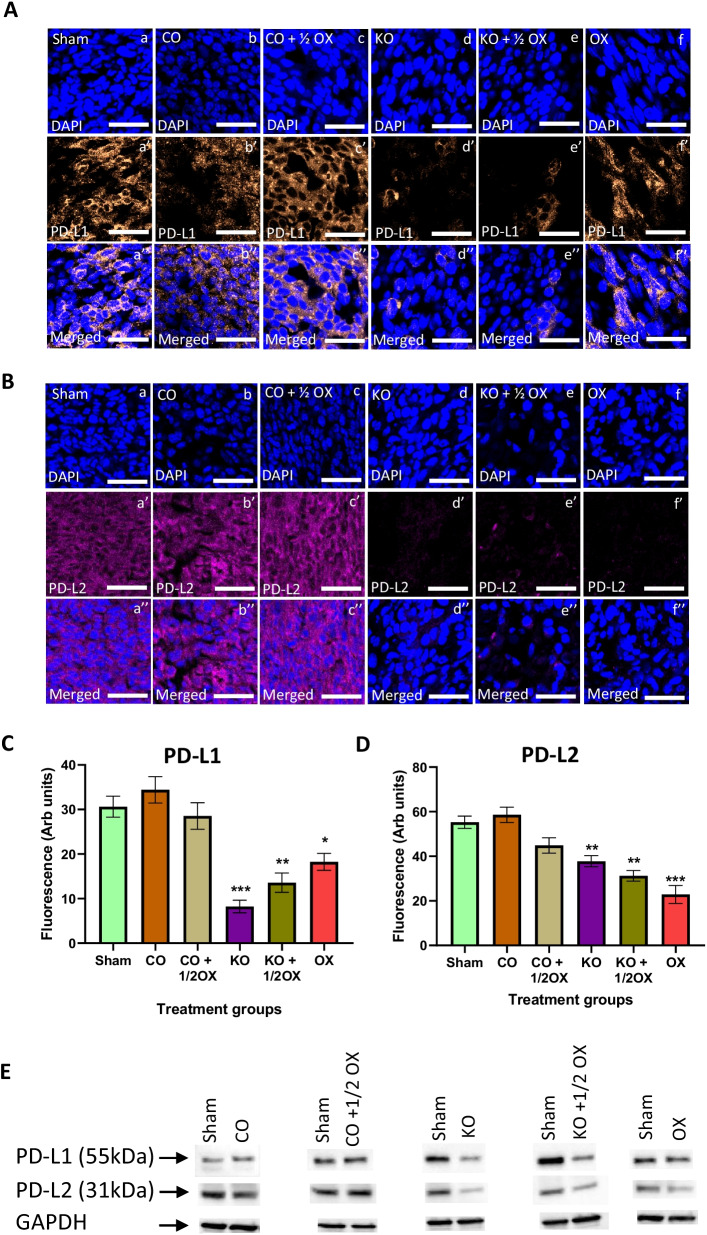
Expression of PD-L1 and PD-L2 following treatments. The expression of PD-L1 (**A**) and PD-L2 (**B**) in tumours following the treatments determined immunohistochemically using monoclonal antibodies. Quantitative analysis of the fluorescence intensity of PD-L1 (**C**) and PD-L2 (**D**) expression in tumour tissues following various treatments determined by immunohistochemistry. Scale bar = 50 µM. Magnification = 40X. N = 6 mice per group. The level of fluorescence was measured using 8 randomly taken images that provide a total area of 1 mm^2^. The results are expressed as mean ± SEM; *P < 0.05, **P < 0.01, ***P < 0.001 compared to the sham group. **E** The expression of PD-L1 and PD-L2 measured by western blotting in tumours following various treatments

The animals treated with KO, KO combined with ½ dose of oxaliplatin, and a full dose of oxaliplatin showed a significant reduction of PD-L1 expression in tumours by 57.7% (*P* < 0.001), 46.7% (*P* < 0.01), and 35.3% (*P* < 0.05), respectively, compared to the sham group (Fig. 5A, C) There were no significant differences in the expression of PD-L1 between animals received treatments with KO, KO combined with ½ dose of oxaliplatin, and a full dose of oxaliplatin (*P* > 0.05). Similar results were observed in the expression of PD-L2 following treatments with KO alone, KO combined with ½ dose of oxaliplatin, and a full dose of oxaliplatin with a reduction by 37.6% (*P* < 0.01), 43.5% (*P* < 0.01), and 58.6% (*P* < 0.001), respectively, compared to the sham animals (Fig. 5B, D). The expression of PD-L2 was not significantly different between the animals that received treatments with KO, KO combined with ½ dose of oxaliplatin, and a full dose of oxaliplatin (*P* > 0.05). These results were further confirmed by the western blotting assay as shown in Fig. 5E.

**Fig. 6:**
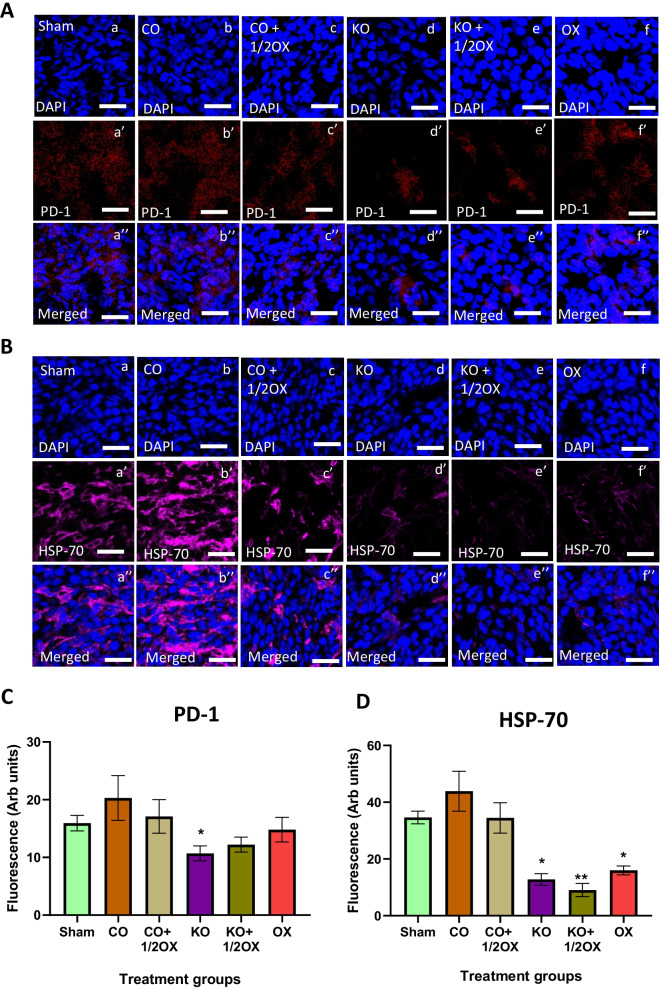
Expression of PD-1 and HSP-70 following treatments. The expression of PD-1 (**A**) and HSP-70 (**B**) in tumours following various treatments determined immunohistochemically using monoclonal antibodies. Quantitative analysis of the fluorescence intensity of PD-1 (**C**) and HSP-70 (**D**) expression in tumour tissues following various treatments determined by immunohistochemistry. Scale bar = 50 µM. Magnification = 40X. N = 6 mice per group. The level of fluorescence was measured using 8 randomly taken images per specimen at 40 × magnification that provide a total area of 1 mm^2^. The results are expressed as mean ± SEM; *P < 0.05, **P < 0.01 compared to the sham group

The animals treated with KO showed a significant reduction of PD-1 expression (*P* < 0.05) in tumours compared to the sham group (Fig. 6A, C). The animals treated with KO, KO combined with ½ dose of oxaliplatin, and a full dose of oxaliplatin showed a significant reduction of HSP-70 level (*P* < 0.05 to *P* < 0.01) in the tumours compared to the sham group (Fig. 6B, D).

## Discussion

This is the first animal study investigating the anti-cancer efficacy of KO supplementation on CRC in comparison with the currently used chemotherapeutic agent, oxaliplatin. This study demonstrated that treatments with KO and KO combined with a ½ dose of oxaliplatin increased the survival rate of mice with CRC in a similar manner as the full dose of oxaliplatin. The results showed that treatment with KO alone significantly reduced tumour weight by 69% and volume by 70%, while KO combined with a ½ dose of oxaliplatin reduced tumour weight by 84% and volume by 83%. More significantly, we found that KO combined with a ½ dose of oxaliplatin reduced tumour size and metastasis at a similar rate with no noticeable side effects compared to the full dose of oxaliplatin where animals showed a reduced appetite, lethargy and weakness. Oxaliplatin-induced side effects observed in this study are consistent with previous reports from our and other groups [8, 9, 34, 35]. In addition, this study showed that treatments with KO, KO combined with a ½ dose of oxaliplatin, and a full dose of oxaliplatin resulted in a high level of expression of cytochrome *c* released from mitochondria in the tumours. These treatments also increased the expression of cleaved caspase-9 and cleaved caspase-3, and DNA damage in tumours. Furthermore, the expression of PD-L1, PD-L2 and HSP-70 was reduced significantly in the tumour tissue of mice received those three treatments. The lower expression of PD-1 was also observed in the animals treated with KO.

Gastrointestinal and neurological complications are well documented as side effects resulting from oxaliplatin treatment in CRC [36]. To overcome these issues, one of the most common practices is dose or frequency reduction with chemotherapy treatment. This could provide a better adaptation to the chemotherapeutic drug with fewer side effects and could encourage patients to continue treatment for a longer period until the desired results are obtained [34]. The fact that animals received the combined treatment of KO with a ½ dose of oxaliplatin increased survival rate and reduced the tumour growth comparably with the full dose of oxaliplatin but without side effects, as demonstrated in this study, suggests a possible interaction of KO and the chemotherapeutic drug. It also implicates the potential role of KO as an adjunctive agent to reduce the side effects associated with oxaliplatin treatment.

KO is a rich source of LC n-3 PUFA, EPA and DHA, as well as antioxidant astaxanthin [11]. Previous studies have investigated the effects of LC n-3 PUFA or fish oil combined with chemotherapeutic agents in the treatment of cancer [35], and identified that combined treatments of LC n-3 PUFA with chemotherapy decreased the required dose of chemotherapeutic agents, hence the side effects associated with higher doses of chemotherapy. The study by Vasudevan et al. [37] reported that EPA administered together with a combination of 5-fluorouracil (5-FU) and oxaliplatin (FuOX) greatly reduced tumour growth in mice with HCT-116 and HT-29 cell xenografts. The authors observed that the reduction in the size of tumour was primarily due to the decrease of tumour cell proliferation attributed to increased sensitivity of tumour cells to the EPA/FuOx combined therapy. Rani et al. [38] investigated the effects of treatment with fish oil combined with 5-FU on CRC and reported that it significantly reduced tumour burden and the 5-FU-associated side effects in a model of DMH/DSS-induced colon cancer in Balb/c mice. The authors also observed that fish oil supplementation increased the effectiveness of 5-FU and activated both intrinsic and extrinsic apoptotic pathways in these mice. Based on this evidence, it is likely that LC n-3 PUFA play an important role in the beneficial effects of KO combined with a ½ dose of oxaliplatin observed in the present study.

Astaxanthin is one of the most common carotenoids and has displayed more powerful anti-oxidative properties than other carotenoids. It exerts several important functions in the human body including prevention of oxidation of EPA and DHA, modulation of exacerbated inflammation responses, and control of carcinogenic processes [39, 40]. Previous studies have discovered the beneficial effects of astaxanthin as a therapeutic agent for various diseases without toxicity or side effects. It also showed preclinical anti-tumour effectiveness both in vivo and in vitro [41] in different cancer models such as oral cancer [42], colon cancer [43], bladder cancer [44] and leukemia [45]. The association of astaxanthin with the beneficial effects of KO combined with a ½ dose of oxaliplatin is not clear in the present study. It is possible that both LC n-3 PUFA and astaxanthin in KO play a role. Further animal studies including co-supplementations of isolated bioactive components including EPA, DHA, and astaxanthin combined with oxaliplatin are warranted to understand their specific contribution to the overall anti-cancer properties of KO in vivo.

Cytochrome *c* (Cyt *c*) is a peripheral protein located in the inner membrane of the mitochondria, and it functions as an electron transporter in-between complex iii and complex iv in the respiratory chain [46]. It is produced in the cytosol as an apoprotein and then translocated to the mitochondria. The majority of Cyt *c* is loosely attached to the intermembrane in mitochondria, and the remaining 15% are tightly bound to the inner membrane through electrostatic and hydrophobic interactions [47]. If cells receive apoptotic stimuli in the presence of ATP, Cyt *c* is released from the mitochondria by opening the pores of the outer membrane. Cyt *c* then binds to apoptosis protease activating factor 1 (Apaf-1) and generates an apoptosome complex [48]. The activated apoptosome can facilitate autocatalytic activation of caspase-9 essential for the initiation of intrinsic apoptosis through stimulation of caspase-3 [49]. The present study found that mice received treatments with KO, KO combined with a ½ dose of oxaliplatin, and the full dose of oxaliplatin had a significantly higher expression of Cyt *c* in tumour tissues. These treatments also increased significantly the level of cleaved caspase-9 and cleaved caspase-3 in tumour tissues. The animals treated with KO alone showed the highest level of cleaved caspase-9 and cleaved caspase-3, followed by those treated with KO combined with a ½ oxaliplatin. These results are consistent with our in vitro observations on CRC cell lines following the treatment with FFAE of KO [19, 20]. The present data provide in vivo evidence that inhibition of CRC tumour growth by KO may occur through the activation of intrinsic mitochondrial death pathway involving the increased release of Cyt *c* and activation of caspase-9 and caspase-3 leading to tumour cell death. These mechanistic impacts of KO are more likely related to its LC n-3 PUFA as demonstrated in our previous in vitro studies [20], although the role of astaxanthin is not clear.

Our results presented in Fig. 4 demonstrate a significantly high level of DNA damage in groups treated with KO alone, KO + ½ OX, and OX alone confirmed by measurement of 8-hydroxy-2'- Deoxyguanosine (8-OHdG). The interaction of reactive oxygen species with the nucleobases of the DNA strand, such as guanine, leads to the formation of 8-OHdG. Based on this evidence, 8-OHdG has been widely used as a biomarker of oxidative stress-induced DNA damage, and measurement of 8-OHdG level is commonly used to evaluate a load of oxidative stress [50]. Our results demonstrate that the level of DNA damage is similar between three treatment groups, KO, KO + ½ OX, and OX, although other markers of oxidative stress-induced apoptosis, Cyt *c* and cleaved caspase 9, are lower in the oxaliplatin-treated group compared to the KO-treated group. These data suggest that the observed DNA damage in the oxaliplatin-treated group involves other mechanisms apart from the intrinsic mitochondrial apoptotic pathway studied in the present study.

Oxaliplatin exerts its cytotoxic effects mainly through DNA damage leading to apoptosis of cancer cells. DNA damage induced by oxaliplatin can be caused by the formation of DNA lesions due to platinum–DNA adducts, arrest of G1 and G2 phases of the cell cycle, inhibition of mRNA synthesis, and induction of immunogenic cell death [51, 52]. In clinical settings, oxaliplatin is combined with other cytotoxic therapeutic agents with varying degrees of success and severe side effects [9]. Our study demonstrates for the first time that KO supplementation enhances cytotoxic effects of oxaliplatin by augmenting apoptosis of cancer cells via the intrinsic mitochondrial apoptotic pathway. Other studies demonstrated that oxaliplatin induces extrinsic mitochondrial apoptotic pathway via caspase 8 activation in HT-29 colorectal cell line [53]. In addition, the low level of caspase-3 in the oxaliplatin-treated group observed in our study suggests that oxaliplatin might have induced non-apoptotic cell death. Oxaliplatin is a potent inhibitor of survivin, a member of the IAP (inhibitor of apoptosis) family, as shown in colorectal cancer cell lines SW480, DLD1, and HT29 [54]. Dysfunction of survivin induces mitotic catastrophe, a form of non-apoptotic cell death caused by aberrant mitosis and eventually causes slow cell death [55]. Further studies are warranted to elucidate whether KO supplementation augments molecular pathways involved in oxaliplatin-induced apoptotic and non-apoptotic cell death.

PD-L1 and PD-L2 are members of the B7 family of molecules and ligands for PD-1 receptors, which are expressed on activated T and B cells [56]. These two ligands are commonly expressed on T and B cells, dendritic cells, and macrophages, as well as a variety of tumour cells, including CRC cells [57]. The interaction of PD-L1 and PD-L2 with the PD-1 receptor on T cells inhibits T cell proliferation, cytokine production, migration cell adhesion, and finally induces apoptosis [22]. Previous studies showed that blocking of the PD-L1/PD-L2 and PD-1 interaction can reestablish the T cell function and can mediate anti-tumour activity by reducing cancer cell proliferation and migration [58]. Our previous in vitro study showed that the FFAE of KO suppresses the expression of PD-L1 in human CRC cells [24]. The present study is the first demonstrating that KO supplementation downregulates the expression of PD-L1 and PD-L2 in the tumour tissues from mice with orthotopic CRC in the same manner as a full dose of oxaliplatin. Further studies are required to validate the results observed in this research. Furthermore, the reduction of PD-1 expression in the tumour tissues indicates the enhancement of a pre-existing anti-tumor immune activity due to KO treatment. We also found a reduction in the level of HSP-70 in the tumours from mice treated with KO, KO combined with a ½ dose of oxaliplatin, and the full dose of oxaliplatin. No studies on the effects of KO and n-3 PUFA on the expression of HSP-70 in CRC have been performed previously. HSPs are the most abundant cellular proteins involved in protein stability [59]. The studies found that HSPs are highly expressed in various types of cancer, and that leads to cancer cell proliferation, differentiation, invasion and metastasis [25]. HSP-70 is a stress response protein that can inhibit the apoptosis of cancer cells. Furthermore, the overexpression of HSP70 is shown to increase tumorigenicity [25, 60]. Taken together, our results indicate that KO has the potential to suppress CRC development through the inhibition of PD-L1/PD-L2 interaction with PD-1 and HSP-70.

Based on the findings from this study, supplementations with 150 g/kg of KO, and KO combined with ½ dose of oxaliplatin resulted in significant benefits against CRC in the mouse model with no noticeable side effects. Clinical trials are warranted to determine the efficacy and dosage of this natural product in the prevention and attenuation of CRC in humans.

## Conclusion

This study showed that KO supplementation significantly reduced colorectal tumour growth in vivo compared to sham treatment. Combined treatment with KO and ½ dose of oxaliplatin exhibited a significant anti-tumour effect and reduced tumour metastasis at a similar rate as the full dose of oxaliplatin without adverse side effects. The tumor-suppressive effect of KO observed in this in vivo study is likely associated with its anti-proliferative, apoptotic, and anti-inflammatory functions via activation of the intrinsic mitochondrial death pathway. These findings provide preclinical evidence for the anti-cancer properties of KO and its potential role as an effective adjunctive therapy for CRC.

## Data Availability

The datasets used and/or analysed during the current study are available from the corresponding author on reasonable request.

## Abbreviations

Apaf-1: Apoptosis protease activating factor 1
ATCC: American tissue culture collection
CO: Corn oil
CRC: Colorectal cancer
Cyt *c*: Cytochrome *c*
DAPI: 4,6-Diamidino-2-phenylindole
DHA: Docosahexaenoic acid
DNA: Deoxyribonucleic acid
EPA: Eicosapentaenoic acid
FCS: Foetal calf serum
FFA: Free fatty acids
FFAE of KO: Free fatty acids extractions of KO
GAPDH: Glyceraldehydes-3-phosphate de-hydrogenase
GI: Gastrointestinal
H&E: Haematoxylin and Eosin
HEPES: 4-2-Hydroxyethyl-1-piperazineethanesulfonic
HEPES: 4-2-Hydroxyethyl-1-piperazineethanesulfonic
HSP-70: Heat shock protein-70
KO: Krill oil
LC n-3 PUFA: Long chain n-3 poly unsaturated fatty acid
OCT: Optimal cutting temperature compound
OX/OXAL: Oxaliplatin
PBS: Phosphate-buffered saline
PBS-T: Phosphate-buffered saline + Tween 20
PD-1: Programmed death receptor
PD-L1: Programmed death ligand-1
PD-L2: Programmed death ligand-2
PUFA: Polyunsaturated fatty acids
RIPA: Radioimmunoprecipitation assay buffer
RNA: Ribonucleic acid
SD: Standard deviation
SDS: Sodium dodecyl sulphate
SEM: Standard error of mean
